# Impact of All-Terrain Vehicle Accidents on Pediatric Patient Outcomes at an Appalachian Children’s Hospital

**DOI:** 10.7759/cureus.27726

**Published:** 2022-08-06

**Authors:** Elizabeth Mannino, Patricia L Chambers, Hannah Wheeler, Seth Brown, Bracken Burns, Matthew Leonard

**Affiliations:** 1 Trauma Surgery, Johnson City Medical Center, East Tennessee State University, Johnson City, USA; 2 Pediatric Emergency Medicine, Niswonger Children's Hospital, Quillen College of Medicine, Johnson City, USA; 3 Trauma/Research, Johnson City Medical Center, Johnson City, USA; 4 Pediatric Emergency Medicine, Niswonger Children's Hospital, Johnson City, USA; 5 Surgery, East Tennessee State University - Quillen College of Medicine, Johnson City, USA; 6 Trauma, Johnson City Medical Center, Johnson City, USA

**Keywords:** all-terrain vehicles, atv, helmet use, pediatric atv, trauma pediatric

## Abstract

Introduction

The use of all-terrain vehicles (ATVs) has become increasingly popular as an outdoor recreational activity among people living in the United States, particularly in areas such as the southeast. There are significant risks involved with riding ATVs, especially in the pediatric population, due to lack of training and experience. The purpose of this study was to evaluate the outcomes of pediatric patients involved in ATV-associated accidents.

Methods

This study is a retrospective review of 98 pediatric patients ages 15 years and younger involved in ATV accidents who were admitted to a pediatric hospital between January 2015 and December 2020. Outcomes, including types of injuries sustained, length of hospital stay, length of ICU stay, and injury severity score (ISS) were analyzed between age groups (0-5, 6-10, and 11-15).

Results

The mean hospital stay across all age groups was 1.7 ± 1.9 days, mean ICU stay was 3.8 ± 4.0 days, and mean injury severity score (ISS) was 5.9 ±4.8. The 11-15-year-old age group had a significantly longer hospital stay and higher ISS scores compared to both of the younger age groups (0-5 and 6-10 years old). There was no difference in ICU days between the age groups. Orthopedic injuries were the most common type of injury, occurring in 55% of all patients, followed by head injuries in 29% of patients, and spinal fractures in 2% of patients. The most common orthopedic fracture in the 11-15-year-old group was tibia/fibula, while humerus fractures were the most common type of fracture in the 0-5 and 6-10 year age groups. Orthopedic procedures were required in 35% of all included patients. There was no statistically significant difference in types of injuries and types of fractures sustained between each group. Chest injuries, including pneumothorax, lung contusions, and rib fractures, occurred most often in the older age group 11-15 years (n=65). Those who experienced chest injuries had a higher ISS, although it was not statistically significant (p=0.06) compared to those who did not have chest injuries. There was no difference in hospital or ICU length of stay in patients with chest injuries.

Conclusions

The results of this study demonstrate the outcomes of pediatric patients admitted for ATV accidents at a rural Appalachian pediatric hospital and provide an overview of the most common injuries involved in this trauma mechanism. Pediatric patients aged 11-15 years of age involved in ATV accidents are at risk for longer hospital length of stay and higher ISS compared to younger age groups. Additionally, patients ages 11-15 were more susceptible to chest injuries following ATV accidents. The results of this study will be used to develop a standardized trauma protocol for the management of this specific trauma mechanism in the pediatric population based on common injury patterns among each age group.

## Introduction

All-terrain vehicles (ATVs) have become an increasingly popular activity in the United States, specifically in Appalachian regions, including East Tennessee. There are approximately 100 pediatric ATV-related fatalities yearly in the United States. This includes over 30,000 emergency department visits annually for pediatric-related ATV accidents, with medical costs approaching one billion dollars [1]. Pediatric ATV trauma can vary between $322 to $310,435 [2]. A literature review by Denning et al. (2018) describes the major risk factors of pediatric ATV trauma, which include lack of training, carrying passengers, lack of helmets, and riding off-road. Extremity injuries are among the most common injuries nationwide. However, the leading causes of death include brain injuries and multi-organ trauma [1]. Although uncommon, genitourinary trauma associated with ATV accidents has been reported. Renal trauma with associated splenic and liver lacerations has been reported in pediatric ATV trauma [3]. Comparisons between pediatric and adult ATV trauma have demonstrated pediatric riders are more likely to sustain more severe injury patterns [4].

Studies have been conducted previously to delineate outcomes of both adult and pediatric ATV-associated trauma in the Appalachian region. Testerman (2009) conducted a retrospective review at a level one trauma center in East Tennessee and collected data over a ten-year period to determine the number and severity of adult and pediatric ATV admissions. The data was compared to data from an earlier period to determine if the number of ATV admissions had increased. Testerman found that overall, ATV crash injury patients had increased by 78% during this period; however, pediatric ATV crash injury patients (age 16 years and younger) decreased during this time. The age of injury trended towards older age groups. He found that the injury severity and type of injuries had not changed from an earlier time. In this particular study, 96% of patients were not wearing helmets prior to their accident [5].

There have been several other studies that focused specifically on pediatric-associated ATV trauma in other areas of the country, including Pennsylvania and middle Tennessee [6,7]. Several studies have been published that focused on specific types of injuries associated with pediatric ATV accidents. For example, Shannon et al. (2018) conducted a retrospective study that evaluated orthopedic injuries from snowmobile, dirt bike, and ATV accidents in a pediatric population [8]. Mangano et al. (2006) conducted a retrospective review of patients at St. Louis Children’s Hospital that experienced neurosurgical injuries after an ATV-associated collision [9].

Based on our literature review, there have been no published studies focused on pediatric-specific ATV trauma in the Appalachian region. This region includes East Tennessee, Southeastern Kentucky, and Southwest Virginia. The goal of this study was to evaluate injury types and outcomes of pediatric patients involved in ATV accidents in the catchment area of our Level one adult trauma center. Additionally, we planned to compare the results of this study to other studies that have been published regarding pediatric ATV trauma to determine if the outcomes were consistent between the studies. Our final goal is to encourage marketing campaigns to raise awareness of the risk of injuries associated with pediatric ATV trauma in the Appalachian region, specifically in the age groups that tend to be the most susceptible to serious injuries. We hope to provide this information to the hospital system marketing team to eventually create an injury prevention programs that could be advertised within the community. We hypothesized that the outcomes of pediatric ATV trauma would be similar to previous studies.

## Materials and methods

This study is a retrospective review of ninety-eight pediatric patients ages 15 years and younger involved in ATV accidents who were admitted to a pediatric hospital between January 2015 and December 2020. The study was conducted at Johnson City Medical Center in Johnson City, Tennessee. Inclusion criteria included age less than 15 years old and admission to the pediatric hospital due to injury caused secondary to an ATV collision. Exclusion criteria included age greater than 15 years old and ATV collision not resulting in injury or admission to a pediatric hospital. All data were collected during this time period and entered into a secured trauma patient database. Demographic and pre-hospital data were collected, including age, gender, method of transportation, helmet use, and whether the patient was the driver or the passenger of the ATV. Outcome data points included the types of injuries sustained (orthopedic, neurosurgical, and chest trauma), length of hospital stay, length of ICU stay, average ventilator days, injury severity score (ISS), need for surgical intervention, mortality, and disposition. The patients were divided into three age groups: 0-5, 6-10, and 11-15. Comparisons were made between the three age groups to determine differences, if any, between pre-hospital factors and outcomes (and specific injuries) following the accident. Excel version 2108 (Microsoft, Redmond, Washington) and JASP version 0.16.1 (JASP Team, Bow, Washington) were used for descriptive and statistical analysis. T-tests were used to analyze total hospital and intensive care unit (ICU) stay and ISS between the age groups. Chi-squared testing was used to analyze types of injuries and fractures.

## Results

**Figure 1:**
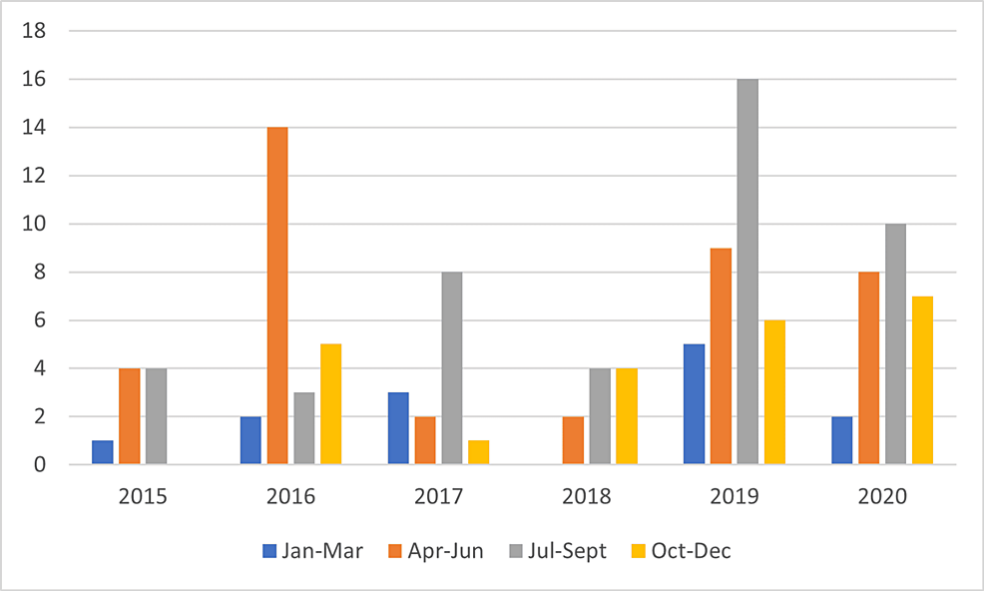
Number of pediatric patients admitted following ATV accidents based on time of year ATV  - all-terrain vehicle

Ninety-eight pediatric patients, 15 years and younger (average age 10.9 ± 4.0 years), were admitted for all-terrain vehicle accidents between January 2015 and December 2020. The number of pediatric admissions was further divided by month and year. The greatest number of admissions throughout the five-year study period were typically during April through September (Figure 1).

Mean hospital days were 1.7 ± 1.9 days, mean ICU days were 3.8 ± 4.0 days, and mean injury severity score was 5.9 ±4.8 (Table 1).

**Table 1:** Length of hospital days, ICU days, and injury severity score by age group ISS - injury severity score

Variable	0-5 years	6-10 years	11-15 years	p-value
Hospital days	1.15	1.2	1.9*	≤0.05
ICU days	2	1.5	4.7	
ISS	4.15	4.1	6.8*	≤0.05

Forty-eight patients arrived at the hospital by ambulance, 17 by helicopter, 20 by personal car, and 13 lacked documentation of mode of transport to the hospital. Over half (54%) of patients were documented as not wearing a helmet (n=53), 25 patients (26%) stated that they were wearing a helmet, and 20 patients had no documentation of helmet use. Seventy of the patients were the driver of the ATV, 27 were the passenger, and one was of unknown status. There was no statistically significant difference noted in the number of hospital days, number of ICU days, and ISS between the drivers and the passengers. The injured patients were predominately male (66 of 98; 67%), between the age of 11 and 15 years (65 of 98; 66%), and 60% (58 of 98) sustained at least one fracture. Orthopedic injuries were the most common type of injury, occurring in 55% of pediatric patients, followed by head injuries in 28 patients (29%). Spinal injuries, rib fractures, and internal injuries were most common in the 11-15 age groups (Table 2).

**Table 2:** Types of injuries by age group

Type of injury	0-5 years	6-10 years	11-15 years
Upper extremity fracture	6	5	17
Lower extremity fracture	3	4	17
Facial fracture	1	1	6
Head injury/concussion	3	4	21
Internal injuries	-	-	6
Burns	-	1	1
Rib fractures	-	-	3
Spinal injuries	-	-	8

Humerus fractures (13; 48%) and radius/ulna fractures (8; 30%) were the most common upper extremity fracture. Tibia/fibula fractures (13; 54%) and femur fractures (8; 33%) were the most common lower extremity fractures (Table 3).

**Table 3:** Types of orthopedic fracture by age group

Type of fracture	0-5 years	6-10 years	11-15 years
Humerus	4	5	4
Radius/ulna	1	-	7
Femur	1	2	5
Tibia/fibula	1	1	11
Clavicle	1	-	1
Scapula	-	-	1
Pubis	-	1	-
Acetabulum	-	1	-
Metacarpal	-	-	3
Metatarsal	-	-	1

Eight patients sustained spinal fractures (2% of total fractures). Thirty-five percent (n=34) of patients with extremity fractures required orthopedic operative interventions. All but one patient were able to discharge home. One patient required transfer to another inpatient facility.

A sub-analysis was done to investigate differences in outcomes among the various age groups (0-5, 6-10, and 11-15). The 11-15-year-old age group had a statistically significant longer length of stay and higher ISS scores compared to both of the younger age groups. There was no difference in the length of ICU stay (Table 1). One patient out of the total population was ventilated for five days. The most common type of fracture in the 0-5 and 6-10-year age groups was humerus fractures. The most common fracture in the 11-15-year-old group was the tibia/fibula. There was no significant difference in types of injuries and types of fractures sustained between each group. There were eight patients with chest injuries, including pneumothorax, lung contusions, and/or rib fractures. The average age of patients with chest injuries was 13.9 years. Those patients who experienced chest injuries had a higher ISS, although it was not significant (p=0.06, 10.6 versus 5.4) compared to those who did not. There was no difference in hospital or ICU length of stay in patients who sustained chest trauma.

## Discussion

The Appalachian region is home to many trails, which include ATV trails in the mountains. ATVs are not just used for common farm use but also a significant source of recreation. A mountainous area can mean terrain is more difficult to drive on, often being uneven rocky terrain. Testerman (2009) studied a pediatric and adult cohort of 300 patients involved in ATV-associated accidents in East Tennessee, Southwest Virginia, and Southeast Kentucky. His study focused on similar factors to our study, including patient demographics, helmet use, injury type, injury severity score, hospital length of stay, ICU length of stay, ventilator days, and discharge disposition, among a variety of other factors. The study demonstrated an increase over time in the number of ATV-related injuries requiring admission to the level one trauma center. The study also demonstrated a decrease in the number of pediatric-associated ATV injuries during the same time period [5]. Our study showed an increase in pediatric patient admissions following ATV accidents during our study period. Of note, there was a two-hospital system merger that occurred in 2018, which may have contributed to a higher total number of admissions in 2019 and 2020. Our study also demonstrated that males ages 11-15 had statistically significant longer length of stay and higher injury severity scores when compared to both the younger age groups. Denning et al. (2014) conducted a study comparing the characteristics and determinants of fatal pediatric ATV trauma. This study demonstrated that patients in the 12 to 15-year-old age group accounted for more than half of the pediatric ATV-related fatalities [10]. Although our study did not have fatalities, the 11-15 age group did have higher injury severity scores when compared to the younger groups suggesting that older pediatric groups continue to be the at most risk.

Garay et al. (2017) published a multi-institutional study in Pennsylvania evaluating pediatric-related ATV accidents [6]. The results from this study were consistent with our study, with significant hospital length of stay and ISS. Their values were slightly higher than ours. However, ours utilizes mean values rather than median values. The majority of patients (55%) included in Garay et al.’s study had at least one bone fracture below the cervical spine, which is also consistent with our study. Unni et al. (2012) conducted a retrospective review of pediatric ATV-associated trauma and had a similar sample size (n=163) to our study which also demonstrated similar results. Approximately 64% of the study population were in the 10-15-year-old age group. Helmets were used only in 33% of patients, which is consistent with our data. The Unni study further stratified injuries associated with helmet use and found that helmet use resulted in fewer injuries to the head, neck, and face [7].

Denning et al. (2012) conducted a retrospective review and comparison of off-road and on-road ATV accidents in Iowa over seven years. This cohort of patients included both adults and pediatric patients. Approximately 30% of the cohort at each location included pediatric patients. This study demonstrated that regardless of the location of the accident, helmets were associated with a reduced risk for the number and severity of helmet use. The study demonstrated the necessity that laws be created that restrict ATV road use and the importance of reinforcement of these laws. This is a potential direction that could be studied in the future with the Appalachian patient population [10].

Vittetoe et al. (2022) examined socioeconomic factors associated with helmet use in pediatric patients involved in ATV and dirt bike trauma. Data from this study demonstrated that unhelmeted riders were older than helmeted riders, with a median age of 13. There was a greater number of males than females who did not wear helmets and a greater number of patients overall who did not wear helmets. The unhelmeted riders had a higher ISS compared to helmeted riders. This is consistent with our study that demonstrates that most patients in our cohort did not wear helmets [11]. However, we did not find a significant difference in ISS between helmeted versus unhelmeted patients.

One of the main limitations of our study is the inability to further stratify certain aspects of the subjects due to the de-identification of data. For example, we did not analyze the correlation between helmet use and head trauma. Other limitations include the small sample size of 98 patients over five years and the single-institution data source. The results would likely be more reproducible if this was a multi-institution study including a larger sample size. We also did not observe soft tissue injuries in the data set. Looking at these injuries in future studies could be beneficial. 

Future directions of this study include creating a larger sample size spanning a longer period. Additionally, we hope to add multiple institutions in the area to the study. Due to the nature of the terrain in the region and inexperienced young riders, looking at riders in the Appalachian region versus a rural community in a region without mountains could yield some interesting findings. Finally, just as Jessula et al. (2017) compared the frequency, nature, and severity of pediatric ATV trauma before and after a marketing campaign aimed at increasing awareness surrounding pediatric ATV trauma in Nova Scotia, the results of this study can be used to create a marketing campaign to raise awareness for children, parents, and educators regarding safety surrounding ATV and helmet use [12]. A follow-up study could be done to determine if a significant difference in age, length of hospital stay, and helmet use changes. 

## Conclusions

There was an increase in pediatric ATV-related injuries. Patients aged 11-15 years are at higher risk of extended hospital length of stay and higher ISS compared to younger age groups. The majority of our patients had at least one bone fracture, and over half were documented as not wearing a helmet. The results of this study will contribute to the development of an injury prevention program within our community. This will hopefully provide awareness among family members and providers regarding common injury patterns seen in various age groups involved in ATV accidents in our region.
